# Commentary on Whitsel *et al*.: Smoking, alcohol use and the brain‐ the challenge of answering causal questions

**DOI:** 10.1111/add.15802

**Published:** 2022-01-25

**Authors:** Jorien L. Treur

**Affiliations:** ^1^ Department of Psychiatry, Amsterdam UMC University of Amsterdam Amsterdam the Netherlands

**Keywords:** Alcohol, brain, causality, genetic, smoking, triangulation

*Smoking and alcohol use have long been associated with indicators of advancing brain age, but it had been challenging to determine if this is a causal relationship. Carefully designed longitudinal imaging studies and studies that apply genetic causal inference methods are now becoming available, allowing more reliable answers to causal questions*.

Whitsel and colleagues have conducted an elegant longitudinal study, incorporating structural magnetic resonance imaging (MRI) data [1]. Their aim was to test if a history of heavier smoking and alcohol consumption in early mid‐life is associated with more advanced brain ageing later in life (a composite measure of cortical thickness, cortical surface area and subcortical volume). Their findings suggest that, in men, both heavier smoking and alcohol consumption at age 40 years predict advanced brain ageing 16 years later. The authors rightfully note that they cannot establish causality, because their study was observational and they were not able to correct for brain age at baseline. It is therefore interesting to compare these findings to results from research methods that can aid causal inferences. In particular, Mendelian randomization (MR) is an increasingly popular method that employs a set of genetic variants highly predictive of a proposed risk factor, e.g. smoking or alcohol use, as instrumental variables to test if there are causal effects on an outcome [2]. To conduct an MR study, effect estimates of the genetic variants on risk factor and on outcome are obtained from genome‐wide association studies (GWAS). Now that large MRI GWAS are available it has become feasible to perform MR to investigate causal questions pertaining to brain measures.

A number of relevant MR studies have been published very recently. The first was a study in which the authors conducted a GWAS of white matter hyperintensities—a radiological trait associated with brain ageing—and additionally performed MR [3]. Their findings provide evidence that smoking causally increases the occurrence of white matter hyperintensities, suggesting that smoking accelerates brain ageing (alcohol was not studied). Another MR study investigated effects of alcohol consumption and hazardous alcohol use on a composite measure of predicted brain age [4]. They found consistent evidence that higher alcohol consumption causally increases brain age, and limited evidence that alcohol use disorder has similar effects (with the cautionary note that the latter was largely driven by a single genetic variant). Finally, a third MR study investigated both smoking and alcohol use in relation to subcortical brain volume, and found evidence to suggest that there are causal decreasing effects (on amygdala and hippocampal volume for alcohol and on hippocampal volume only for smoking) [5]. Combined, these MR studies seem to bolster the findings of Whitsel and colleagues.

However, much is still unclear and important (causal) questions remain. For instance, what is the role of polysubstance use? None of the MR studies investigated smoking and alcohol combined in a multivariable model, while we know that their use is correlated [6]. In addition, cannabis has not been taken into account in any of the studies described above, while its use is correlated strongly with smoking [7]. It would be valuable to conduct multivariable MR, which allows the inclusion of multiple risk factors, to test effects of different substances together. Another worthwhile direction is to link the tentative evidence for causal effects of smoking on brain structure to the hypothesis that smoking could be a causal risk factor for mental health disorders [8, 9]. If smoking causally affects brain structure, it seems plausible that this could lead to adverse changes in brain function and subsequently mental health problems [10]. However, studies that explicitly test (mediation) pathways from smoking to brain structure and then mental health are lacking.

Unravelling the complex relationship of smoking and alcohol use with brain structure and function is challenging. However, given the potential benefits for prevention and treatment, it is worth increasing our efforts to do so. This may be most pressing for smoking, as it is not widely recognized that smoking could have a negative effect on the brain. In some cases it is even the opposite—health professionals are often hesitant to urge people with a mental health disorder to give up smoking, fearing that quitting might worsen their mental health problems [11]. In reality, this is probably the group that could benefit most from smoking cessation [12]. Going forward, it will be crucial to ‘triangulate’ findings from multiple approaches to gain a clearer understanding of how smoking and alcohol use affect the brain [13, 14]. If different methods, with different strengths and weaknesses, point in the same direction, this would provide robust evidence that can be acted upon—e.g. by guiding policy decisions or aiding the design of public health messages.

## DECLARATION OF INTERESTS

None.
